# Poly(vinyl chloride) Films Incorporated with Antioxidant ZnO-Flavonoid Nanoparticles: A Strategy for Food Preservation

**DOI:** 10.3390/foods13172745

**Published:** 2024-08-29

**Authors:** Lilian R. Braga, Maria Graciele Oliveira, Leonardo M. Pérez, Ellen T. Rangel, Fabricio Machado

**Affiliations:** 1 Laboratório de Desenvolvimento de Processos Químicos, Instituto de Química, Universidade de Brasília, Campus Universitário Darcy Ribeiro, Brasília 70904-970, DF, Brazil; graciele.oliveiradf@gmail.com (M.G.O.); ellen.tanusrangel@gmail.com (E.T.R.); fmachado@unb.br (F.M.); 2 Facultad de Ciencias Bioquímicas y Farmacéuticas, Universidad Nacional de Rosario (UNR), Suipacha 570, Rosario S2002LRL, Sant Fe, Argentina; lperez@fbioyf.unr.edu.ar; 3 Facultad de Química e Ingeniería del Rosario, Pontificia Universidad Católica Argentina (UCA), Av. Pellegrini 3314, Rosario S2002QEO, Santa Fe, Argentina

**Keywords:** antioxidant films, active packaging, zinc oxide nanoparticles, quercetin, morin, high-fat food preservation

## Abstract

Antioxidant films were prepared using poly(vinyl chloride) (PVC) incorporated with 0.5% or 1.0% zinc oxide (ZnO)-flavonoid (quercetin or morin) nanoparticles (NPZnO-Q% or NPZnO-M%) via the casting method. NP incorporation within the polymer matrix influenced the structural, morphological, optical, and thermal properties of the PVC-based films, as well as their antioxidant activity as assessed using the DPPH radical scavenging method. Our results indicated that increasing ZnO-flavonoid NP concentration increased films thickness, while reducing ultraviolet light (UV) transmittance but conserving transparency. The presence of NPZnO-Q% or NPZnO-M% improved the surface uniformity and thermal stability of the active films. In terms of antioxidant activity, there was an enhancement in the DPPH radical scavenging capacity (PVC/ZnO-Q1.0% > PVC/ZnO-Q0.5% > PVC/ZnO-M0.5% > PVC/ZnO-M1.0% > PVC), suggesting that the packaging can help protect food from oxidative processes. Therefore, these antioxidant films represent an innovative strategy for using as active food packaging material, especially intended for aiding in quality preservation and extending the shelf life of fatty foods.

## 1. Introduction

In recent years, there has been increasing interest in the development of packaging materials that enhance food quality and safety, as well as in methods that delay or minimize food degradation. Lipid oxidation is one of the primary causes of spoilage in fat-rich foods like meat, milk, cereals, and other products, leading to the generation of rancid or off-flavors. In addition, food exposure to light further accelerates this deleterious process [1].

To reduce food lipid oxidation different strategies have been implemented, such as the direct incorporation of synthetic antioxidants and the use of a modified atmosphere in conjunction with high oxygen barrier packaging. Butyl hydroxytoluene (BHT), butyl hydroxyanisole (BHA), tert-butyl hydroxyquinone (TBHQ), and propyl gallate (PG) are among the synthetic antioxidants most frequently used in foods [2]. However, these compounds have raised safety concerns due to potential health risks, including heart diseases and carcinogenic properties. As an alternative, replacing synthetic antioxidants with natural ones has proven to be a suitable and more sustainable strategy to minimize these risks. Among natural antioxidants, phenolic compounds (e.g., α-tocopherol, quercetin, morin, and catechin) and organic acids (e.g., ascorbic acid and citric acid) stand out, as well as plant extracts and essential oils [3,4,5,6].

Quercetin is one of the most effective natural antioxidants due to its great capacity to donate electrons [7]. This bioflavonoid prevents the formation of free radicals through three main mechanisms: (a) by reacting with the superoxide ion; (b) by inhibiting metal-induced hydroxyl radical formation acting as a metal-chelating agent; and (c) by impeding food lipid peroxidation by reacting with peroxyl radicals. The cyclic structures on the quercetin molecule contain -OH groups in specific positions (3, 5, 7, 3′, and 4′) that stabilize free radicals [8,9].

Morin is another natural flavonoid with antibacterial, anticancer, and antioxidant properties [10,11]. It has been suggested that morin can eliminate OH radicals and superoxide anions which are highly reactive during the first stage of lipid peroxidation. Moreover, morin inhibits xanthine oxidase, an enzyme that generates free radicals, by either activating or modifying its structure [11]. It is also known for its metal-chelating property [9].

In recent decades, the development of films incorporating antioxidant compounds acting as food packaging materials has been extensively studied [12]. This active packaging increases food shelf life while maintaining organoleptic properties. However, it is important to notice that films intended to be in contact with foods must adhere to standards and regulations regarding maximum allowable concentrations of antioxidants [13,14]. In this sense, new advances have been made for achieving the safe incorporation of antioxidants [7,13,15] into film polymeric matrices.

It is worth highlighting the casting method, which involves mixing an organic solvent with the polymer and, if necessary, a plasticizer. After solubilization, the mixture is poured onto a plate to allow the solvent to evaporate, and forming a film, which is then removed. The quality of the film depends on polymer cohesion, manufacturing parameters, and the type of plasticizer used. This method is simple, cost-effective, and widely used in research laboratories for producing homogeneous films [16,17].

On the other hand, metallic nanoparticles (NPs) have unique antimicrobial and physicochemical properties which have favored their application into polymeric matrices to provide microbicidal activity against a wide range of microorganisms, including bacteria, fungi, and viruses [18]. Silver, gold, titanium oxide, zinc oxide (ZnO), and copper oxide NPs have proved to be highly versatile for improving packaging properties in different composite materials [18,19,20,21,22,23]. In particular, ZnO NPs display effective antibacterial and antifungal properties [23,24] good thermal stability, and resistance to UV radiation [25]. Considering that zinc is a mineral used in food supplementation and NPs can be safely incorporated into food packaging, the use of ZnO NPs within regulated limits can be advantageous for preserving food quality. However, the use of ZnO NPs in food is subject to varying regulations worldwide, primarily due to safety concerns and the unique properties of these nanoparticles [26]. In the United States, ZnO NPs are classified as ‘generally recognized as safe’ (GRAS) by the Food and Drug Administration (FDA) for certain applications [27]. Allan et al.’s [26] study highlighted the efforts of regulatory bodies across ten countries, including the European Union, through the Global Coalition for Regulatory Science (GCRSR), which aims to enhance regulations and bolster scientific research on the safety and efficacy of nanotechnology in consumer food products. In the study by Tankhiwale and Bajpai [28], ZnO NPs were synthesized in situ on starch-coated polyethylene film, and their antimicrobial activity was evaluated. Besides their antibacterial properties, ZnO NPs offer a more cost-effective alternative compared to other nanoparticles such as gold and silver.

On the other hand, the choice of the polymer is another important factor in the success of active packaging. For a synthetic polymer to be suitable for food applications, some desired properties such as being excellent oxygen and water barrier, and having high transparency, good mechanical resistance, and ease of handling must be accomplished. Among them, polypropylene (PP), linear low-density polyethylene (LDPE), poly(ethylene terephthalate) (PET), poly(ethylene-co-vinyl alcohol) (EVOH), poly(ethylene-acetate) co-vinyl) (EVA), and poly(vinyl chloride) (PVC) [3,6,22,29] have been extensively used. PVC is one of the most widely used thermoplastics globally and is considered a significant commodity. Recognized for its excellent properties such as chemical resistance, inertness, affordability, low flammability, and ease of processing, PVC finds applications in various sectors including medical and hospital materials, the food industry, and other industries [17,30,31]. Given the above, the aim of this study was to develop PVC films incorporating ZnO NPs and the bioflavonoids quercetin and morin, and to investigate their impact on the physicochemical, mechanical, thermal, antioxidant, and optical properties of the synthesized films.

## 2. Materials and Methods

### 2.1. Chemicals

Poly(vinyl chloride) (PVC) with an average molar mass of 130 kDa was kindly provided by the Brazilian company Braskem S.A. (Braskem, Camaçari, Bahia, Brazil) Quercetin hydrate and morin hydrate (Sigma-Aldrich, Darmstadt, Germany), tetrahydrofuran (THF) (Tedia, Fairfield, CT, USA), and 2,2-Diphenyl-1-picrylhydrazyl (DPPH) (Sigma-Aldrich, Darmstadt, Germany) were purchased from suppliers.

### 2.2. Synthesis of ZnO-Flavonoid NPs

**Figure 1:**
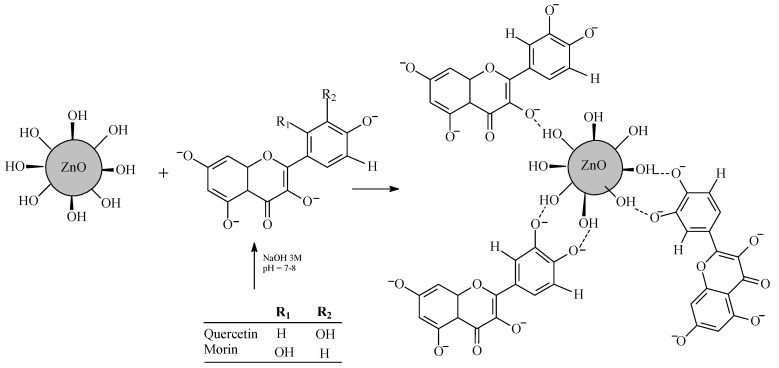
A simplified scheme outlining the steps involved in the synthesis of ZnO-quercetin or ZnO-morin nanoparticles (adapted from Mallakpour and Javadpour [15]).

The NPs containing both ZnO and natural flavonoids (i.e., quercetin or morin) were synthesized as reported by Mallakpour and Javadpour [15] with adaptations. Briefly, for the synthesis of ZnO NPs, 0.5000 g of ZnO was weighed in an analytical balance. Then, 50.0 mL of water/ethanol (8:2 *v*/*v*) was added and the mixture was sonicated for 1 h at room temperature. Subsequently, 0.0125 g of quercetin or morin (1:3 ZnO:flavonoid) was weighed, and 750 µL of methanol was used to solubilize the antioxidant. NaOH solution (3 mol L^−1^) was used to adjust the pH of the suspension between 7 and 8. The suspension was stirred for 3 h and then allowed to stand for an additional 3 h. Finally, the mixture was incubated for 24 h at 65 °C to form ZnO-quercetin or ZnO-morin NPs (NPZnO-Q% or NPZnO-M%), as illustrated in Figure 1.

### 2.3. Production of NP-Incorporating Films

The films were produced using the casting method described by Amar and Parisi [32] with the modifications proposed by Braga et al. [22]. Initially, 0.5 g of PVC was dissolved in 20 mL of THF containing 100 µL of plasticizer (epoxidized soybean oil). The mixture was kept at 60 °C under constant stirring. Then, ZnO-flavonoid NPs (NPZnO-Q% or NPZnO-M%) at concentrations of 0.5% and 1.0% were incorporated into the homogeneous mixture. Subsequently, the mixture was poured onto previously cleaned and sanitized glass plates (approximately 30 × 20 cm) and allowed to dry at 25 °C in the dark for 24 h until the solvent completely evaporated, resulting in film formation (PVC/ZnO-Q0.5%, PVC/ZnO-Q1.0%, PVC/ZnO-M0.5%, and PVC/ZnO-M1.0%). In parallel, a control film (PVC control) without NP addition was prepared under identical conditions.

### 2.4. Caracterization of ZnO-Flavonoid-Containing NPs

Surface images of the films were observed using scanning electron microscopy (SEM) in a JSM-7001F microscope (JEOL Ltd., Akishima, Tokyo, Japan). Each film was fixed onto a sample holder and coated with gold under an inert argon atmosphere for 1 h [17]. The size and shape of the ZnO-flavonoid NPs incorporated into the films was observed using transmission electron microscopy (TEM) in a JEM-1011 microscope (JEOL Corp., Japan). For sample preparation, PVC-based films were dissolved in THF, and a drop of the suspension was deposited onto a grid using a Pasteur pipette. After solvent evaporation, images were taken following the protocol outlined by Mallakpour and Mashal [15].

The light transmittance of the films was measured in the ultraviolet and visible range (200–600 nm) following the method described by Ramos et al. [33] using a Cary 8454 spectrophotometer (Agilent Technologies, Santa Clara, CA, USA). Briefly, film samples were cut into rectangular pieces (2 × 4 cm) and positioned near the inner wall of a spectrophotometric quartz cell and the background was empty accessory. The relative transparency of the films was calculated as the ratio between the absorbance at 600 nm (A600) and the film thickness (measured in millimeters using a micrometer). Determinations were performed in quintuplicate (*n* = 5).

The FTIR-ATR analysis of the antioxidant films was conducted in a Varian^TM^ spectrophotometer (model 640-IR) with attenuated total reflectance (ATR). Measurements were taken in the scanning range from 650 cm^−1^ to 4000 cm^−1^ with a resolution of 4 cm^−1^, accumulating 64 scans, using the empty accessory for the background [17].

Thermogravimetric analyses (TGA) were performed using a DTG-60 analyzer (Shimadzu Scientific Instruments, Marlborough, MA, USA). Film samples (10 mg) were pre-weighed in a platinum crucible and heated from 25 °C to 1000 °C at a rate of 10 °C/min under a nitrogen atmosphere with a flow rate of 30 mL/min [22].

The tensile strength (TS), elongation at break (EB), and Young’s Modulus (YM) of the films were determined using a motorized test frame Multitest 2.5d (Mecmesin, Sterling, VA, USA), equipped with a 100 N digital force gauge. Ten rectangular film strips (60 × 7 mm) were fixed at the ends with double-sided paper tape to prevent tearing during testing. The initial length of the film samples was 30 mm, and the testing velocity was set at 0.05 mm/s. Prior to each tensile test, the samples were conditioned at 25 °C and 58% humidity for 24 h [34].

### 2.5. Antioxidant Activity

The antioxidant activity of the films was determined using the DPPH (2,2-diphenyl-1-picril-hydrazine) method described by Siripatrawan and Harte [35] with some modifications. This method is based on the ability of the antioxidant molecules present in the PVC-based films (i.e., quercetin and morin) to react with the DPPH radical and thus produce a reduction in the absorbance measured at 515 nm. First, the films were cut into small pieces of approximately 0.025 g. Then, the pieces were placed in test tubes with 3 mL of DPPH methanolic solution (0.06 mM) and incubated in the dark at room temperature with shaking. After 3 h and 24 h incubation, the films were removed directly from the solution. The solution was poured into the cuvette without filtration, and the absorbance was measured at 515 nm in a Cary 8454 UV-Vis spectrophotometer (Agilent Technologies, CA, USA). The percentage (%) of antioxidant activity (*Ac*) was calculated according to Equation (1):

(1) $$Ac\;\left(\%\right)=\left(\frac{{Abs}_{DPPH}-{Abs}_{sample}}{{Abs}_{DPPH}}\right)\cdot 100$$

where *Abs_DPPH_* and *Abs_sample_* are the absorbances registered for the DPPH solution and the sample, respectively. Methanol was used as a blank. The results were expressed in *Ac* (%) as the mean value ± standard deviation (S.D.) of three replicates (*n* = 3).

## 3. Results and Discussion

### 3.1. Thickness, UV Light Transmission, and Transparency

The values for thickness, UV light transmission (range 250–400 nm), and transparency (T600) of PVC/ZnO-Q%, and PVC/ZnO-M% with a flavonoid content of 0.5% and 1.0% are presented in Table 1. The incorporation of both ZnO-flavonoid NPs into the PVC polymeric matrix did not result in a statistically significant difference (*p* > 0.05) in film thickness compared to the control PVC film. Thickness is a significant film parameter because various properties (e.g., mechanical, optical, water vapor, and gas permeability) depend on it. As such, there are many factors that affect the structure of film materials. For instance, film thickness can be influenced by high concentrations of solids [36]. In our study, the thickness of the PVC-based films was not affected by the incorporation of ZnO-flavonoid NPs at either of the two concentrations tested (0.5% and 1.0%). In addition, all films synthesized using the casting method showed thickness values similar to commercially available PCV films intended for wrapping foods [37]. In most cases, the selection of a film for packaging is based on its ability to preserve the quality of the food. The storage temperature also influences this selection. Accordingly, PVC bags were effectively used by Gaspar et al. [38] for the storage of guava (a tropical fruit) at 8 °C for 2–3 weeks. In addition, PVC films of 20 μm and 40 µm thickness were used for the storage of fresh-cut fruit at 5 °C and 10 °C [37,39]. Actually, PVC films are currently used to package processed meat [40]. Hence, the thickness values of all PVC-based films obtained here seem suitable for similar applications.

On the other hand, light transmission in the range of 250 nm to 400 nm for PVC control films varied from 78.1 ± 7.3 to 83.8 ± 6.4, indicating poor UV light barrier properties for such films in accordance with the literature [41,42]. Conversely, the reinforcement of PVC films with NPZnO-Q% or NPZnO-M% at 1.0% provided a considerable reduction in the UV transmittance rates. This behavior could be probably due to the light scattering exerted by the presence of a higher amount of ZnO NPs, and the presence of chromophore groups in the flavonoid molecules contributing to UV radiation absorption. A similar observation was reported by Braga et al. [17] in a study of PVC films incorporating Ag-quercetin NPs. Notably, the reduction in UV light transmission exerted by films incorporating ZnO-flavonoids and specially with 1.0% NPs is an essential feature to prevent oxidative rancidity, thereby ensuring food preservation. Oxidative rancidity negatively impacts food quality, causing changes in color, odor, and flavor, as well as reducing the shelf life of the products. Moreover, rancidity represent a problem in the food industry, as it is directly involved in increased food waste and economic losses [43].

**Figure 2:**
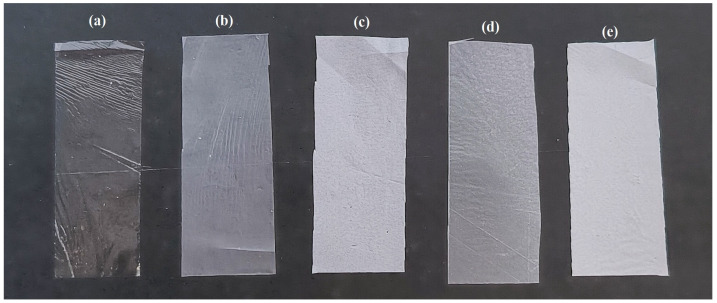
PVC-based films synthesized using the casting method with and without the incorporation of ZnO-flavonoid NPs: (**a**) poly(vinyl chloride) (PVC) (control), (**b**) PVC/ZnO-Q0.5% (**c**), PVC/ZnO-Q1.0%, (**d**) PVC/ZnO-M0.5%, and (**e**) PVC/ZnO-Q1.0%.

On the other hand, PVC control films and those containing 0.5% ZnO-flavonoid NPs exhibited lower transparency values (Table 1). Furthermore, with the incorporation of 1% NPs, there was a further reduction in the transparency of the films, as shown in Table 1 and confirmed by Figure 2, which is consistent with experimental results of studies involving Ag-Cu/LLDPE films [44] and Ag-quercetin/PVC [17]. The slight agglomeration of the nanoparticles due to the increased concentration of ZnO-Q1% is a possible explanation for the transparency reduction. As reported by Emamifar et al. [45], an increase in the amount of ZnO NPs can lead to the formation of agglomerates and the increased heterogeneity of the film. Despite this reduction, the films with 1% NPs may still be suitable for use in the food industry since the primary function of packaging is to protect food against biological, biochemical, and mechanical damages. Therefore, film transparency should be considered secondary to achieving enhanced barrier properties (e.g., antimicrobial effectiveness, and gas and water permeability) and mechanical resistance. However, optical transparency is a valuable feature for consumers since it allows the evaluation of food freshness and appearance through visual inspection. Moreover, transparency is also important for the incorporation of optical sensing systems during food packaging [46].

### 3.2. Morphological Characterization

**Figure 3:**
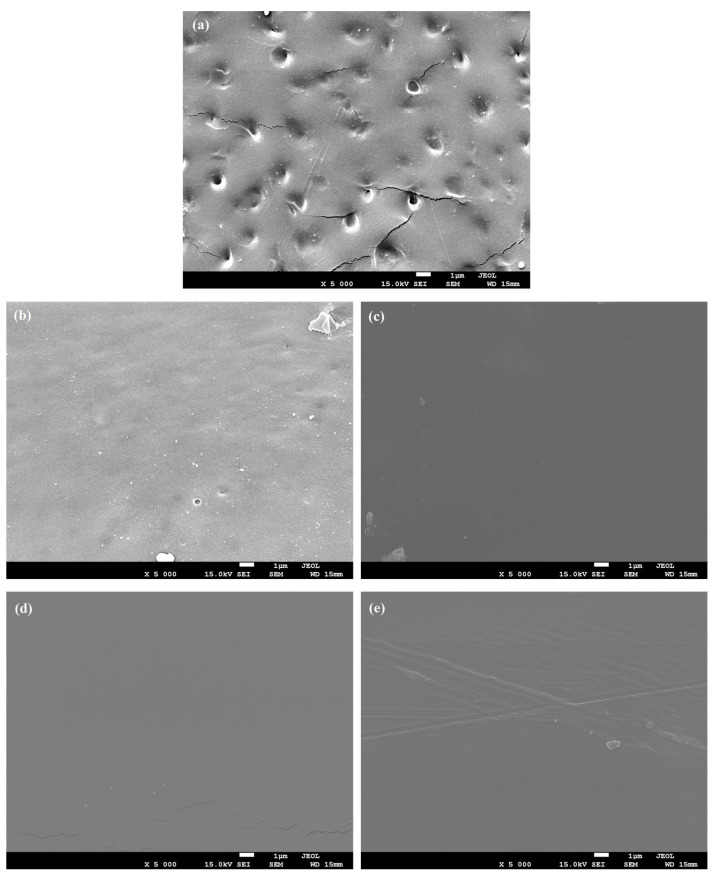
SEM micrographics of film surface (5000× and 15 kv): (**a**) poly(vinyl chloride) (PVC), (**b**) PVC/ZnO-Q0.5% (**c**), PVC/ZnO-Q1.0%, (**d**) PVC/ZnO-M0.5%, and (**e**) PVC/ZnO-Q1.0%.

PVC films exhibited a surface with small pores dispersed within the polymeric matrix, showing irregularities in pore shape and diameter (ranging from 0.5 μm to 0.9 μm) (Figure 3a). However, the incorporation of ZnO-M% and ZnO-Q% NPs into the polymeric matrix (Figure 3b–e) revealed a noticeable change in the film surface, which became smooth, homogeneous, and free of pores. Elashmawi et al. [47] reported in their study on ZnO-NPs in PVC that, due to their small size, the nanoparticles interact more efficiently with the polymer matrix, resulting in good dispersion. These interactions likely fill gaps, smooth the surface, and improve film cohesion, producing a more uniform and less porous surface. Similar findings were reported by Tang et al. [48] in their study on the synthesis of ZnO NPs modified by methacryloxypropyltrimethoxysilane, which were incorporated into PVC. Their study also indicated that ZnO NPs can be homogeneously dispersed in PVC matrices.

It is worth highlighting that the quercetin molecule contains phenolic and pyran rings capable of forming stable and soluble complexes with different metal ions [49]. This likely facilitates a closer interaction with the ZnO NPs, resulting in NP size reduction and promoting stronger chemical interactions with the PVC-based polymeric structure. Due to its chemical structure which is similar to quercetin’s, morin exhibited a comparable behavior (Figure 3d,e).

**Figure 4:**
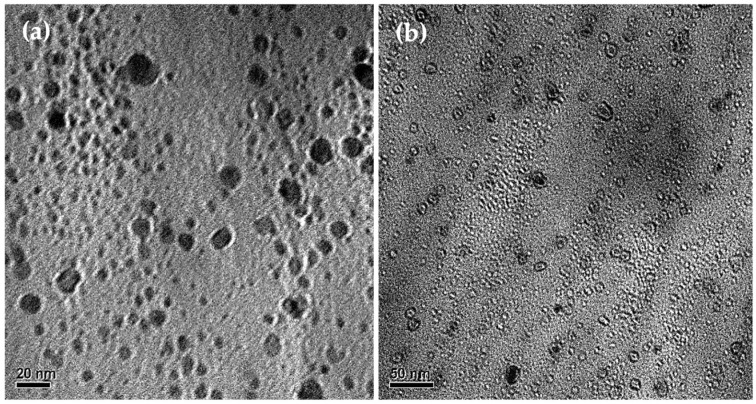
TEM images of ZnO-quercetin nanoparticles obtained after dissolving PVC/ZnO-Q1% films (**a**) scale bar: 20 nm and (**b**) scale bar: 50 nm.

The size of the ZnO-quercetin NPs incorporated into the PVC matrix was confirmed through TEM (Figure 4). As observed, the ZnO-Q1.0% NPs (Figure 4a,b) exhibited a spherical shape, with sizes ranging from 2 to 18 nm.

### 3.3. FTIR-ATR Analysis

**Figure 5:**
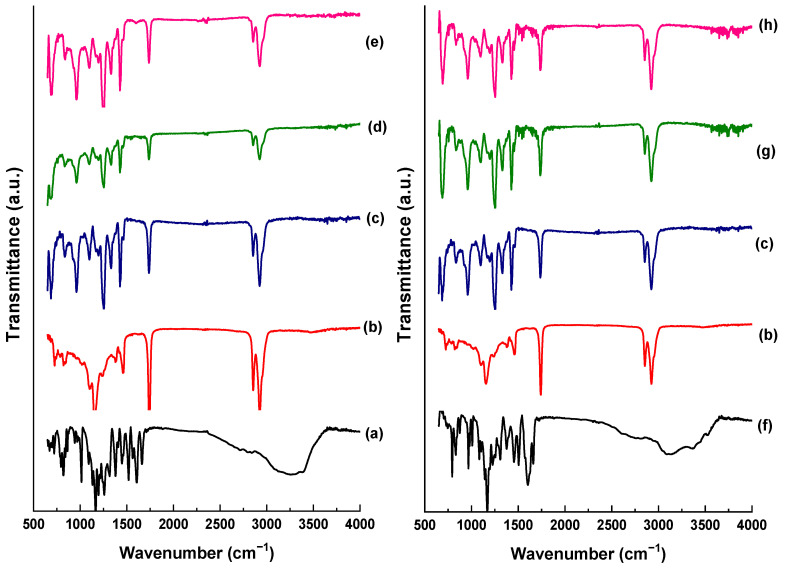
FTIR spectra of films containing ZnO-flavonoid nanoparticles: (**a**) quercetin standard, (**b**) plasticizer, (**c**) poly(vinyl chloride) (PVC) film (control), (**d**) PVC/ZnO-Q0.5% film, (**e**) PVC/ZnO-Q1.0% film, (**f**) morin standard, (**g**) PVC/ZnO-M0.5% film, and (**h**) PVC/ZnO-M1.0% film.

FTIR-ATR analysis was performed to obtain qualitative information regarding the chemical interactions between PVC and the ZnO-flavonoid NPs (Figure 5).

Figure 5a shows the typical bands located at 3000–3500 cm^−1^ of the standard quercetin, corresponding to (OH) bonds. The bands at 1680, 1650, and 1600 cm^−1^ indicate stretching vibrations between (C-C), (C=O), and (C=C), respectively. Similar bands can be observed for morin in Figure 5f, as both molecules belong to the same class of flavonoids and have similar chemical structures.

Figure 5b shows the characteristic bands of the plasticizer (i.e., epoxidized soybean oil) used in the production of all films. The absorption bands located at 2900 cm^−1^ and 2851 cm^−1^ correspond to the asymmetric and symmetrical (C-H) stretching of alkanes (CH_2_), respectively. The 1740 cm^−1^ band corresponds to the (C=O) stretch of aliphatic esters, while the bands at 1460 cm^−1^ and 1380 cm^−1^ are assigned to the C-H bond of aliphatic hydrocarbons in the (CH_2_ and CH_3_) groups, respectively. The band at 1155 cm^−1^ refers to the vibration frequency of the ester group (C-O), and the discrete bands between 800 and 830 cm^−1^ correspond to the epoxy group [50].

Figure 5c shows the characteristic bands of the control PVC film, which agree with those reported in the literature [10]. The bands located at 2800 cm^−1^ and 2915 cm^−1^ correspond to the HCl stretching (CH). At 1250 cm^−1^, there is a prominent band referring to the vibration CH close to the ClCH group [17]. The spectral range between 1000 cm^−1^ and 1100 cm^−1^ shows the C-C characteristic stretching signals. An absorption band located at approximately 966 cm^−1^ can be observed for the methylene group (-CH_2_). The bands extending between 690 cm^−1^ and 650 cm^−1^ are related to the conformational stretching between (C-Cl) [51].

The spectra obtained for the PVC films containing NPZnO-Q0.5% and NPZnO-Q1.0% NPs (Figure 5d and Figure 5e, respectively) displayed slight changes in bands intensities. Furthermore, a shift of the band positioned at 829 cm^−1^ to 820 cm^−1^ was observed, which may indicate the opening of the epoxy ring [52] in the presence of the NPs, forming a complex. Similar trends were observed with the incorporation of NPZnO-M0.5% and NPZnO-M1.0% in the PVC-based polymeric matrix, as shown in Figure 5g and Figure 5h, respectively, with subtle changes in band intensities.

### 3.4. Mechanical Properties

Mechanical property measurements were conducted to determine the strength of the films, which is crucial for maintaining the structural integrity and barrier properties of films intended for food packaging. The results for the tensile strength (TS), elongation at break (EAB), and Young’s Modulus (YM) of the PVC films incorporating ZnO-flavonoid NPs are presented in Table 2.

According to Table 2, all films containing ZnO NPs exhibited a decrease in TS values as the concentration of ZnO-flavonoid (morin or quercetin) NPs increased from 0.5% to 1.0%. This decrease in film strength can be attributed to changes in the intramolecular bonds of the polymer matrix following the addition of a higher amount of ZnO-flavonoids NPs, confirming the reduced TS [53]. Moreover, a reduction in EAB was observed in both PVC films containing ZnO-flavonoid NPs compared to the control PVC films. Das et al. [54] also reported an increase in the deformation percentage and a decrease in the TS of a PVC composite doped with ZnO/Fe, attributing these results to the compatibility (i.e., strong interaction) between PVC and ZnO/Fe.

It is also observed that PVC films incorporating ZnO-morin NPs showed higher TS values compared to both PVC films incorporating ZnO-quercetin NPs and control PVC film. However, in the case of the PVC/ZnO-Q0.5% film, the TS value was not statistically significant different (*p* > 0.05) compared to the control film. Along with a significant increase (*p* < 0.05) in TS, the PVC/ZnO-flavonoid (morin and quercetin) films showed a significant decrease (*p* < 0.05) in EAB values compared to the control PVC films (Table 2), indicating the formation of more resistant and rigid (i.e., less flexible) films. On the other hand, from a statistical point of view, the EAB values observed for the PVC films containing NPs were not significantly affected by the incorporation of ZnO-flavonoid NPs.

Statistically speaking, the effect of ZnO-flavonoid concentration on the YM values was observed only for the PVC films incorporating ZnO-quercetin NPs, which resulted in a decrease from 11.22 ± 0.69 MPa to 6.64 ± 1.29 MPa as the fraction of nanoparticles increased from 0.5% to 1.0%. Moreover, the reduction in the YM observed for both PVC/ZnO-quercetin films (PVC/ZnO-Q0.5% and PVC/ZnO-Q1.0%) also confirmed that the latter are less rigid (i.e., more flexible) than the PVC/ZnO-morin and control PVC films. (N.B. The PVC/ZnO-quercetin films showed no statistically significant difference in YS values when the concentration of ZnO-quercetin NPs was evaluated.) For the PVC/ZnO-M1.0% film, the YS value cannot be considered statistically significantly different (*p* > 0.05) from that of the control film.

Notably, the PVC/ZnO-M0.5% films showed the highest YM values (Table 2). These results could be related to a reduction in the number of attachment points from chain to chain exerted by the morin molecule, letting the PVC-based polymer be deformed without breaking. However, when more ZnO-M NPs are added, this leads to reduced free volume (i.e., the internal space between polymer chains), resulting in a more rigid film [55,56] Hence, the differences in the mechanical properties of the PVC films incorporating ZnO-Q% or ZnO-M% NPs could be related to differences in the structural arrangement, thermochemical, and electronic properties of both bioflavonoids [57]. The incorporation of polyphenols can result in different structural changes and enhanced properties according to the concentration of the flavonoid added, the nature of the chemical groups at the polymeric matrix, and the interactions of the -OH groups of polyphenols [58]. Accordingly, additional studies at a molecular level should be carried out to deepen in these features. However, these analyses are beyond our technical expertise and far from the aim of the present work.

### 3.5. Thermogravimetric Analysis

**Figure 6:**
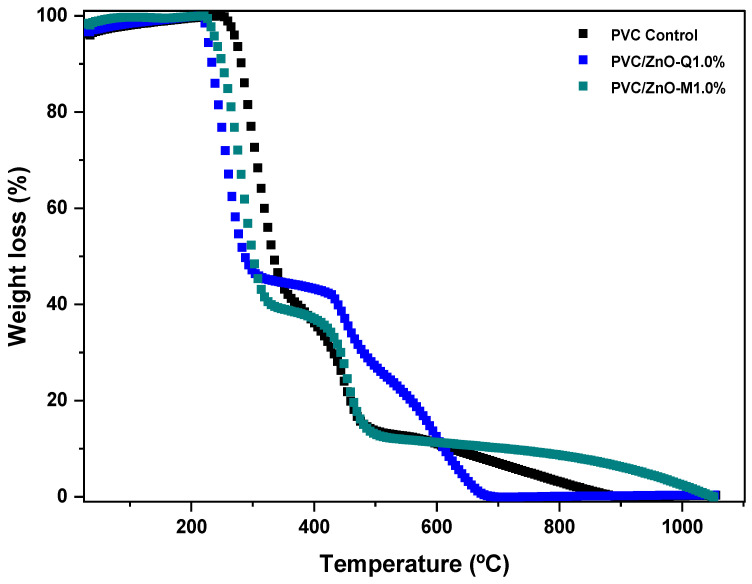
TGA thermograms of poly(vinyl chloride) (PVC) films formulated with or without (control) the incorporation of ZnO-flavonoid nanoparticles.

Figure 6 shows the TGA curves of control and ZnO-flavonoid NP-incorporating PVC films. The thermal degradation of the PVC-based films occurred in two steps. The first stage of degradation started at ~25 °C and concluded at ~200 °C, showing a minimal mass loss of the films, probably due to the evaporation of some residual content of the solvent in the samples [53]. The mass loss observed near 250 °C can be attributed to film dehydration, while the changes in the curve observed between 300 and 400 °C may arise from hydrochlorination, leading to the loss of HCl. Finally, the mass decay between 400 and 500 °C may suggest the degradation of more complex structures on the polymeric chains of PVC with the formation of conjugated polyenes [50]. These results are in line with those obtained by Mallakpour and Jarahiyan [15] for a PVC film incorporating CuO NPs infused with citric acid and ascorbic acid.

The incorporation of ZnO-flavonoid NPs into the PVC matrix led to an increase in film degradation temperature, particularly notable in the case of PVC/ZnO-Q1.0%. These results suggest an improvement in the thermal stability of the developed films. Thermal stability is a key design feature that determines the suitability of a given material for specific applications, as it is related to the material’s ability to retain its original properties (e.g., mechanical) when exposed to heat. This is particularly important in the food industry since the thermal treatment of food is a common procedure for reducing microbiological flora, destroying enzymes that could potentially threaten the consumer’s health, and preserving the quality of the food [59]. In addition to providing information on the thermal stability and decomposition of materials, the test helps to understand how films behave under real storage and transportation conditions. In the study reported by Panea et al. [23], chicken breast meat packaged in LDPE films containing ZnO + Ag nanoparticles showed satisfactory results, with nanoparticle migration within legally permissible limits. Besides delaying chicken breast deterioration, the film reduced lipid oxidation and inhibited bacterial growth on the surface, thereby extending the shelf life. This approach represents a viable alternative to directly applying antioxidants to food.

### 3.6. Antioxidant Activity of Active Films

The deterioration of food during storage induces a sequence of unfavorable alterations in its sensory and quality attributes which contribute to food deterioration and economic losses. Integrating antioxidants into food packaging materials to manage the oxidation of fatty components and pigments can aid in preserving shelf life and maintaining product quality [60].

**Figure 7:**
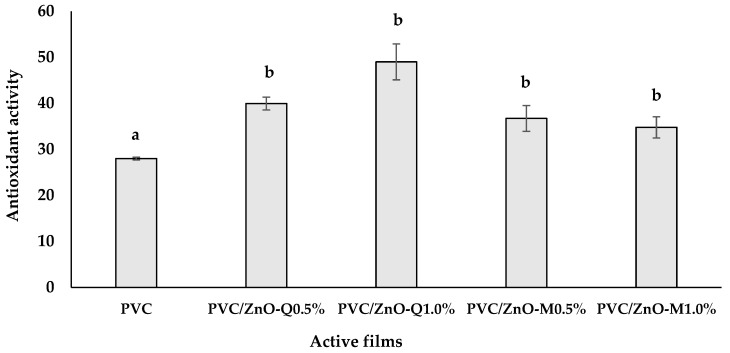
The percentage of antioxidant activity in the active films. Error bars represent the standard deviation (*n* = 3) and different letters mean statistically significant differences (*p* < 0.05).

Figure 7 shows that PVC films incorporating ZnO-flavonoid NPs exhibited a significantly higher DPPH radical scavenging potential compared to the control PVC films. This was expected since the capacity of flavonoids to act as antioxidants has been well documented. However, it was observed that the control PVC film also presented antioxidant activity, probably due to the presence of the plasticizer (epoxidized soybean oil), which is used to prevent the thermo-oxidative degradation of PVC. Moreover, important structure−activity relationships of flavonoid antioxidant activity have also been established [61]. In this sense, the superior performance of PVC/ZnO-Q% films is likely due to the chemical structure of quercetin. This compound belongs to the broader category of polyphenols, specifically those containing hydroxyl groups in the B-ring. According to Masek and Chrzescijanska [62], these hydroxyl groups play a significant role in quercetin’s antioxidant activity by facilitating the transfer of hydrogen atoms to the DPPH radical, and consequently reducing it. This factor becomes crucial in delaying the oxidation process. Although morin has a chemical structure very similar to quercetin’s, with numerous hydroxyl groups (particularly at the C3 position which is responsible for morin’s antioxidant activity), the hydroxyl groups on the quercetin B-ring could have contributed to its greater antioxidant activity compared to morin [63].

Our results shown that both PVC-based films containing ZnO-flavonoid NPs conserve the antioxidant capacity, and thus they can be suitable for using as active packaging. Further studies are required to evaluate the effectiveness of the developed films for inhibiting the lipid oxidation of foods with a high-fat content, such as meat [64], cooked ham, cheese, and sausages [17], and chicken breast meat [23].

## 4. Conclusions

In this study, antioxidant PVC films based on ZnO-flavonoid NPs were synthesized simply and fast using the casting method. The incorporation of NPs into the polymer matrix influenced the films’ morphological, structural, optical, thermal, and mechanical properties. The PVC-based films incorporating NPZnO-Q1.0% and NPZnO-M1.0% exhibited uniform surfaces, proper UV light barrier properties, and transparency, all of which are desirable characteristics that are attractive for the food sector. Furthermore, incorporating NPZnO-Q1.0% into the film polymer matrix resulted in more thermally stable films. Moreover, films with NPZnO-Q% were more flexible compared to PVC/NPZnO-M% and PVC control films. Finally, all PVC-based films incorporating the bioflavonoids (i.e., quercetin and morin) demonstrated a higher DPPH radical scavenging potential, providing greater protection against oxidative processes. These results are promising for food packaging applications since the active films developed here can help to prevent the lipid oxidation of fatty foods, conserving food safety and quality.

## Figures and Tables

**Table 1:** Values (mean ± S.D.) of thickness (*n* = 10), UV light transmission (%), and transparency (A600) (*n* = 5) of poly(vinyl chloride)-based films containing ZnO-flavonoid nanoparticles ^‡^.

Films	Thickness (µm)	Wavelength (nm)				
		250	300	350	400	A600
PVC control	25.0 ± 5.3 ^a^	78.1 ± 7.3 ^b^	82.0 ± 6.6 ^b^	83.8 ± 6.4 ^b^	83.0 ± 6.4 ^b^	0.30 ± 0.14 ^a^
PVC/ZnO-Q0.5%	32.0 ± 10.3 ^a^	73.0 ± 5.6 ^b^	72.0 ± 8.7 ^b^	75.1 ± 8.1 ^b^	76.7 ± 8.0 ^b^	0.29 ± 0.11 ^a^
PVC/ZnO-Q1.0%	32.5 ± 8.2 ^a^	1.0 ± 0.4 ^a^	1.8 ± 1.1 ^a^	1.9 ± 1.0	2.6 ± 1.6 ^a^	4.58 ± 0.80 ^b^
PVC/ZnO-M0.5%	34.0 ± 5.2 ^a^	76.7 ± 2.0 ^b^	81.0 ± 1.2 ^b^	83.0 ± 1.6 ^b^	82.0 ± 1.4 ^b^	0.20 ± 0.02 ^a^
PVC/ZnO-M1.0%	32.0 ± 7.9 ^a^	1.2 ± 0.3 ^a^	2.3 ± 2.0 ^a^	3.3 ± 3.0 ^a^	4.5 ± 3.9 ^a^	3.11 ± 1.00 ^b^

^‡^ Values with different letters in each column are significantly different (*p* < 0.0 5).

**Table 2:** Values (mean ± S.D., *n* = 10) of tensile strength (TS), elongation at break (EAB), and Young’s Modulus (YM) of films ^‡^.

Films	TS (MPa)	EAB (%)	YM (MPa)
PVC control	10.86 ± 1.50 ^b^	16.97 ± 3.20 ^b^	6.03 ± 0.78 ^b^
PVC/ZnO-Q0.5%	9.80 ± 1.20 ^b^	8.51 ± 1.07 ^a^	3.74 ± 0.82 ^a^
PVC/ZnO-Q1.0%	5.67 ± 0.45 ^a^	8.69 ± 3.66 ^a^	3.50 ± 0.35 ^a^
PVC/ZnO-M0.5%	21.16 ± 3.24 ^d^	10.52 ± 2.77 ^a^	11.22 ± 0.69 ^c^
PVC/ZnO-M1.0%	13.77 ± 0.98 ^c^	6.86 ± 3.06 ^a^	6.64 ± 1.29 ^b^

^‡^ Values with different letters in each column are significantly different (*p* < 0.05).

## Data Availability

The original contributions presented in the study are included in the article, further inquiries can be directed to the corresponding author.
